# Accuracy of linear-probe ultrasonography in diagnosis of infraorbital rim fractures

**DOI:** 10.1186/s13089-022-00298-y

**Published:** 2023-02-10

**Authors:** Chatchai Pruksapong, Nuttadon Wongprakob, Minth Panphichet

**Affiliations:** 1 Division of Plastic and Reconstructive Surgery Department of Surgery, Phramongkutklao Hospital and College of Medicine, Bangkok, Thailand; 2 Department of Radiology, Phramongkutklao Hospital and College of Medicine, Bangkok, Thailand

**Keywords:** Linear probe ultrasonography, Infraorbital rim fracture, Computerized tomography

## Abstract

**Background:**

Maxillofacial fractures are a common cause of visits to emergency department, accounting for more than 400,000 annual visits in the United States. Gold standard diagnostic tool is conventional computerized tomography (CT) or 3DCT reconstruction. However, the disadvantages of CT are radiation exposure, unavailable in some hospital and expensiveness. Whereas the bony structures overlap is a problem in diagnostic when using plain film X-ray. The objective of this study is to show the accuracy of a linear-probe ultrasound compared to computed tomography and plain film X-ray in diagnosis of infraorbital rim fracture.

**Methods:**

Patients clinically suspected of an inferior orbital rim fracture underwent linear-probe ultrasonographic investigation, plain film X-ray and CT. CT was used as gold standard in this diagnostic study. A radiologist and senior resident of plastic surgery were the examiner and interobserver for comparison.

**Result:**

A total of 34 patients with suspected infraorbital rim fractures were investigated. Sensitivity of the linear-probe ultrasonography versus CT in the detection of infraorbital rim fracture was 92.9% (95% CI 66.1–99.8), specificity was 90.0% (95% CI 68.3–98.8), positive predictive value was 86.7% (95% CI 59.5–98.3), negative predictive value was 94.7% ( 95% CI 74.0–99.9), accuracy 91%.

**Conclusion:**

Linear probe ultrasonography is a good diagnostic tool and has better reliability than the plain film X-ray and can be used as alternative to CT in inferior orbital rim fracture.

## Background

Maxillofacial fractures are a common cause of emergency department (ED) visits, accounting for more than 400,000 annual visits in the United States alone [1], and occurs in approximately 5–33% of patients experiencing severe trauma [2, 3]. The incidence of maxillofacial trauma varies from region to region. Cause of injuries varies by age group, [4] and racial group; type of fractures also depends on the group studied [4, 5]. The most typical site of injury is zygomaticomaxillary complex (38.6%) [6]. 35% of zygomaticomaxillary complex fracture involve infraorbital rim [5, 7].

Clinical presentations of zygomaticomaxillary complex or infraorbital rim fractures are tenderness, periorbital ecchymosis, diplopia, ocular movement limitation, numbness of infraorbital area, nausea and vomiting in children [8–10]. Gold standard diagnostic tool is computerized tomography (CT) [11] by the coronal, axial plane with 1-mm thin-slice 3D reconstruction. However, CT scanning leads to radiation exposure, is expensive and not available in some hospitals. But the overlap of bony structures may not lead to correct diagnosis when using plain film X-ray.

Ultrasonography [12] is a widely available method that can be performed bedside in ED with no radiation exposure, and is inexpensive. Previous studies show the benefits of using ultrasonography as an alternative method for investigating facial fracture [9, 13–15]. Inferior orbital rim fracture can be diagnosed by using curved array ultrasound [16]. However, most of the ultrasound machines which have been used for trauma patients in ED do not have curved array, and mainly use a linear probe.

The objective of this study is to show the accuracy of a linear-probe ultrasound compared to CT and plain film X-ray in diagnosis of infraorbital rim fracture.

## Material and methods

An institutional review board approved the study which was conducted between April of 2019 and May 2021 at Phramongkutklao hospital. Our diagnostic study compared the receiver operating characteristics (sensitivity, specificity, and positive predictive value (PPV) and negative predictive value (NPV)) of linear-probe ultrasound, plain film X-ray and 64-slice CT 3D of facial bone to determine the concordance between them in detecting inferior rim of zygomatic bone fracture.

The inclusion criteria consisted of patients aged 18 years or older who presented with a history of injury and had clinically suspected inferior orbital rim fracture which may include periorbital trauma, periorbital ecchymosis, diplopia, limited ocular movement, periorbital tenderness (especially in the infraorbital rim area), or numbness at infraorbital area. The exclusion criteria are obvious stepping at infraorbital area, other emergency condition such as ruptured globe, history of moderate-to-severe head injury, and unstable vital signs. All the participants provided written informed consent for participation in the study and publication of the photographs.

Our study protocol starts with bedside ultrasound (GE LOGIQ e 2012 with 7.5 MHz linear transducer), which was performed in ED, followed by collection of and data.

**Fig. 1:**
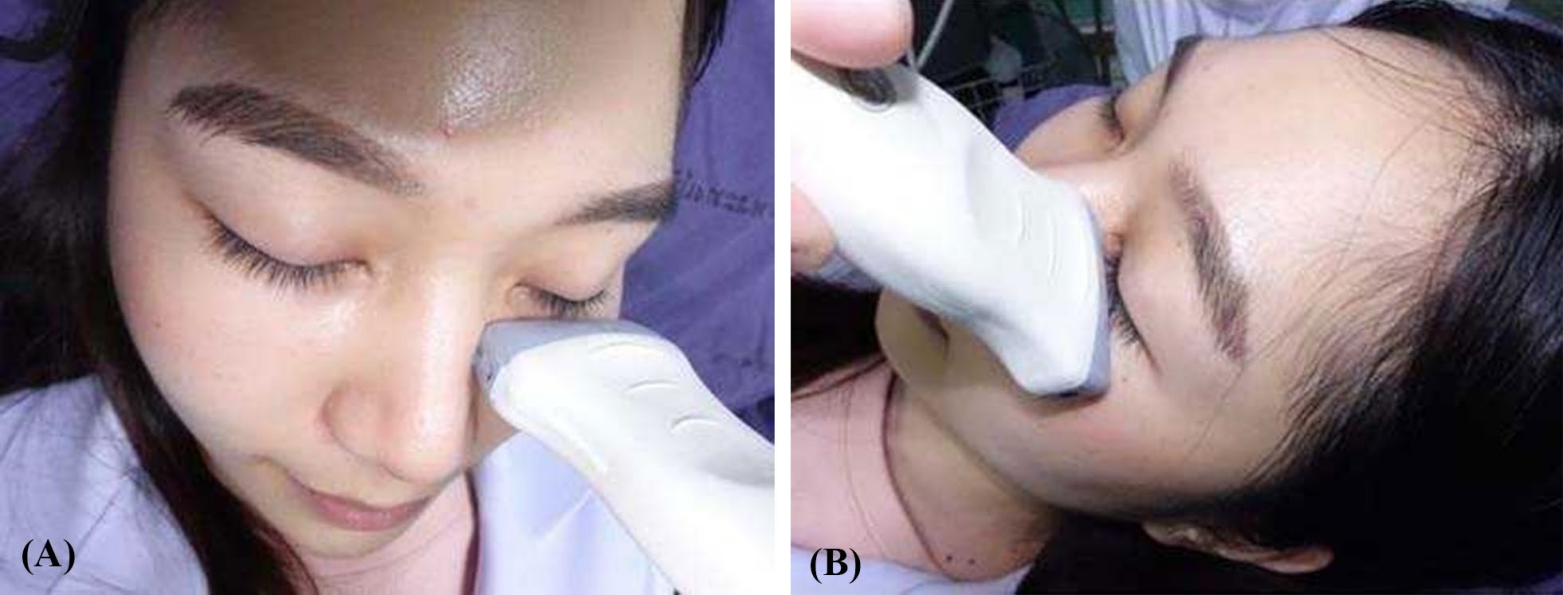
Demonstrating the use of linear-probe ultrasound examination of infra orbital rim. **A** and **B** Demonstration using ultrasound for infraorbital rim examination

**Fig. 2:**
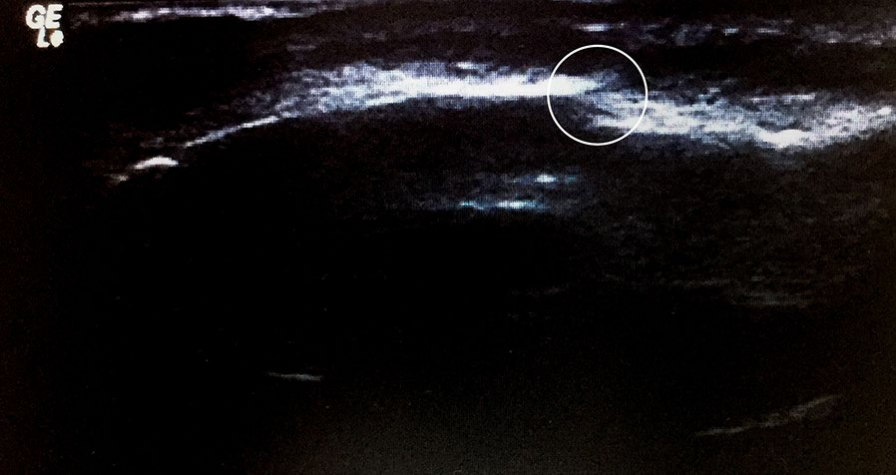
**a**–**b** Demonstration of the discontinuation of infra orbital rim fracture site

**Fig. 3:**
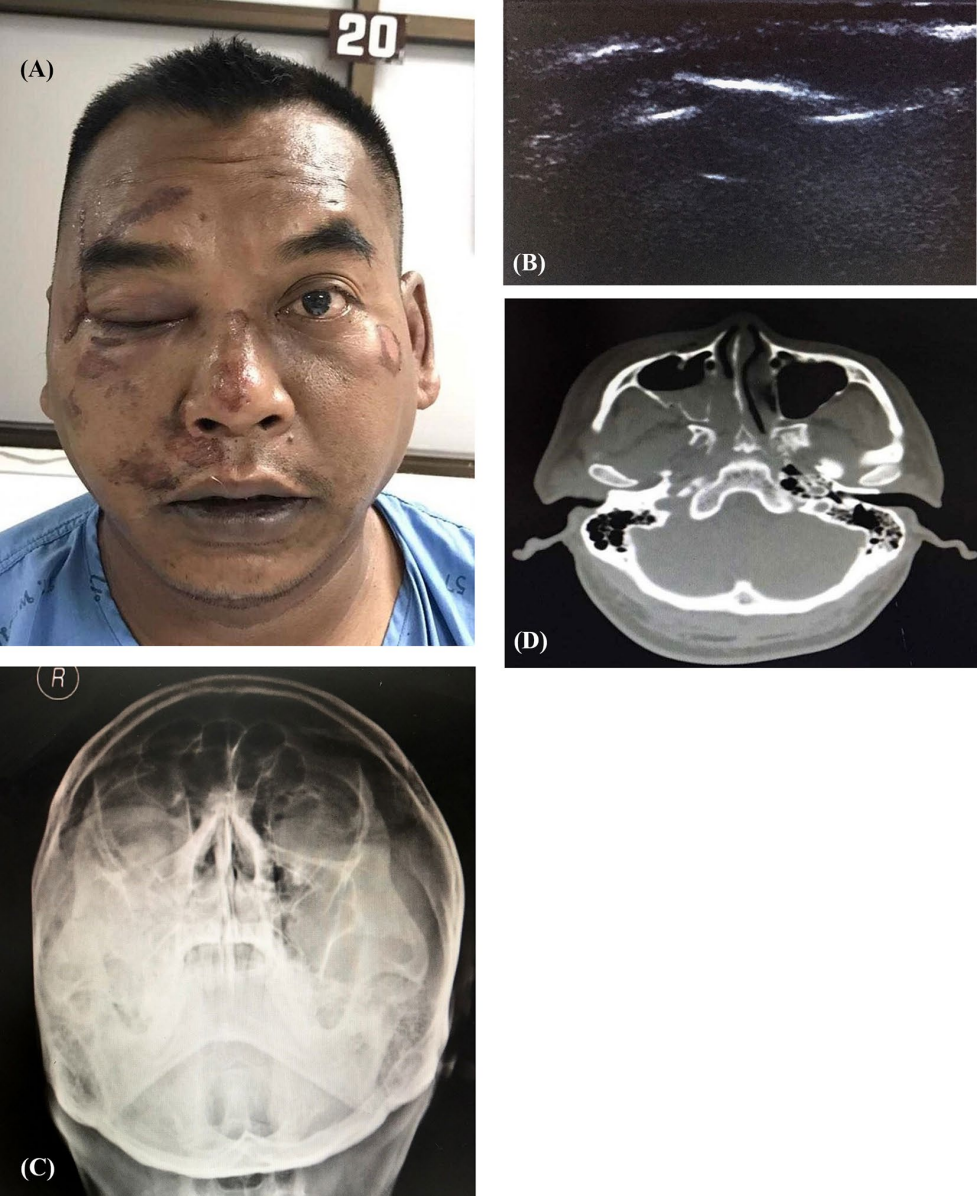
**a**–**d** Case of 42-year-old male: history of vehicle accident, presented with right infraorbital rim tenderness; diagnosed with fracture of right zygoma

**Fig. 4:**
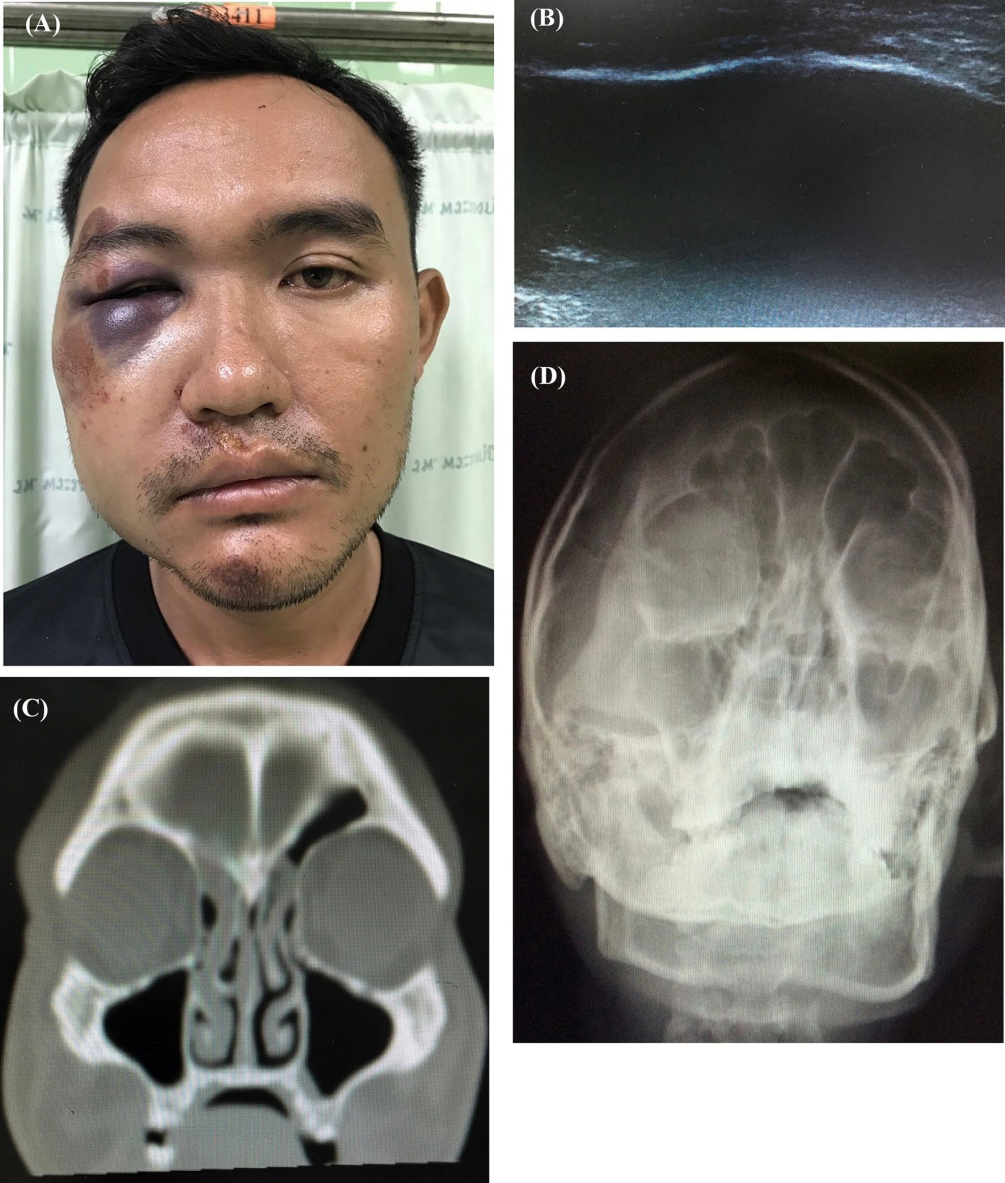
**a**–**d** Case of 33-year-old male: history of vehicle accident, presented with marked swelling of right periorbital area, no fracture of infra orbital rim detected

For the investigation of infraorbital rim fracture, the transducer was positioned at the infraorbital rim and the orbital rim with minimal pressure to look for any fracture line, while the patients were asked to close their eyes (Fig. 1). Plain film X-ray (PA and Waters’ view) and CT of facial bone with 3D bone reconstruction (Canon Lightning Aquilion 64 CT scan thin slice 1 mm.) were accomplished. A “positive case” for each of the methods used was defined as a discontinuity or displacement of bone cortex (Fig. 2). A “negative case” was defined as the absence of discontinuity or displacement of bone cortex. Imaging data of X-ray and ultrasound were interpreted by the senior resident plastic surgeon and staff radiologist, whereas CT was interpreted by staff radiologist before proceeding with the standard treatment (Figs. 3, 4).

The categorical variables were summarized using percentages and frequency of occurrence. Descriptive statistics such as means, medians, ranges, and standard deviations were calculated to derive continuous variables such as sensitivity, specificity, PPV, and NPV of linear transducer ultrasound, plain film X-ray and CT 3D facial bone (set as gold standard) for comparison. P value of < 0.05 was considered significant. All statistical calculations were performed using SPSS version 22 (Chicago, IL).

## Results

Thirty-four patients met the inclusion criteria. The study was completed in all patients. Patients demographics are summarized in Table 1. Most of patients were male (88 percent) and most of accidental cause were vehicle accident (67.6 percent). Most of patients were male (88%) and most of the causes were vehicle accidents (67.6%). Periorbital ecchymosis (88.2%) was the most common clinical appearance. All the patients underwent bedside linear transducer ultrasound, plain film X-ray and CT 3D of facial bone. Ten patients underwent an open reduction and internal fixation; 20 patients were treated conservatively, and four patients underwent a closed reduction.

**Table 1:** Baseline clinical characteristic of 34 patients

Characteristic	N (%)
Sex	
Male	30 (88.2)
Female	4 (11.8)
Age (mean) ± S.D	39.67 ± 19.82
Cause of injury	
Vehicle accident	23 (67.6)
Fall from height	7 (20.6)
Body assaults	3 (8.8)
Other	1 (2.9)
Alcohol use	10 (29.4)
Clinical appearance	
Periorbital ecchymosis	30 (88.2)
Subconjunctival hemorrhage	22 (64.7)
Limited EOM*	9 (26.5)
Tender at inferior orbital rim	23 (67.6)
Stepping	16 (47.1)
Decrease sensation	7 (20.6)

EOM* extra ocular muscle

Sensitivity of the linear-probe ultrasonography, when compared to that of CT in the detection of infraorbital rim fracture was 92.9% (95% CI 66.1–99.8), specificity was 90.0% (95% CI 68.3–98.8), PPV was 86.7% (95% CI 59.5–98.3), NPV was 94.7% (95% CI 74.0–99.9), accuracy 91%. The sensitivity of plain film X-ray when compared to that of CT in the detection of infraorbital rim fracture was only 78% (95% CI 49.2–95.3), specificity was 80% (95% CI 56.3–94.3), PPV was 73.3% (95% CI 44.9–92.3) NPV was 84.2% (95% CI 60.4–96.6), accuracy 79% percent (Table 2).

**Table 2:** Sensitivity and specificity compared to computer tomography

Variable	Plain film (95%CI)	Ultrasound(95%CI)
Sensitivity	78(49.2–95.3)	92.9(66.2–99.8)
Specificity	80(56.3–94.3)	90(68.3–98.8)
Positive predictive value	73.3(44.9–92.3)	86.7(59.5–98.3)
Negative predictive value	84.2(60.4–96.6)	94.7(74–99.9)
Accuracy	79	91

The ultrasound diagnosis performed by the senior resident of plastic surgery and that of the staff of radiology department was compared. Sensitivity of senior resident of plastic surgery was 92.3% (95% CI 47.2–93.3), and that of the radiologist was 92.9% (95% CI 66.2–99.8). Specificity of senior resident of plastic surgery was 87% (95% CI 63.3–97.3), and that of the radiologist was 90% (95% CI 68.3–98.8); PPV of senior resident of plastic surgery was 84% (95% CI 48.6–96.7) and that of the radiologist was 86.7% (95% CI 59.5–98.3); NPV of senior resident of plastic surgery 90.2% (95% CI 69.4–97.6) and that of the radiologist was 94.7% (95% CI 47–99.9); accuracy of senior resident of plastic surgery 88% and that of the radiologist was 91% (Table 3).

**Table 3:** Performance between operators

Variable	Resident plastic surgery (95%CI)	Radiologist (95%CI)
Sensitivity	92.3(47.2–93.3)	92.9(66.2–99.8)
Specificity	87(62.3–97.3)	90(68.3–98.8)
Positive predictive value	84(48.6–96.7)	86.7(59.5–98.3)
Negative predictive value	90.2(69.4–97.6)	94.7(74–99.9)
Accuracy	88	91

## Discussion

Inferior orbital rim is a part of zygomatic and maxillary bone and called as zygomaticomaxillary suture [17], this area contains infraorbital nerve which when injured or involved in fracture causes numbness in the infraorbital area [18]. Inferior orbital rim is one of the most common areas involving maxillofacial fracture; it may be a simple zygomatic bone fracture or severe multiple facial bone fractures or pan facial fracture. Clinical signs of inferior orbital rim fracture include swelling, periorbital ecchymosis, subconjunctival hemorrhage, and numbness [19, 20]. In our study, 88.2% of patients had periorbital ecchymosis, 64.7% had subconjunctival hemorrhage and 20.6% developed reduced sensation in the infraorbital area.

Actually, most definite clinical sign of fracture is clinical stepping. Fracture of the inferior orbital is easy to detect if swelling is not much, but most of patients present with swelling, which makes it difficult to examine and palpate the stepping area. The diagnosis of an orbital fracture is challenging because its clinical presentation usually varies and the anatomy of the region is complex [21–23]. Although the capability of a conventional non-contrast CT in providing multiplanar thin slices or 3D reconstruction of facial bone with good spatial resolution and 3D images in orbital fractures [24–26] makes it the imaging method of choice or accepted as gold standard [27–29], there is a significant concern regarding the hazard of radiation, and in some emergency situations clinical state of patient may not stable enough to be transferred to CT room. Financial issues could also be a matter of concern for some patients.

Ultrasound is a less invasive diagnostic tool with no radiation effect; it consists of high-frequency mechanical vibration not audible to human ear. Cost of ultrasound is also much less than that of CT. Ultrasound can be used in trauma patients, and most emergency departments have ultrasound for detecting cardiac tamponade of pericardial effusion or focus assessment sonography in trauma in abdominal injury. Ultrasound was first used as a diagnostic tool for maxillofacial fracture in 1981 by ORD et al. [30] to detect medial orbital wall fracture. Several authors have reported their studies [16, 31–33]. Most of studies used ultrasound in medial, lateral wall and orbital floor fracture. The least sensitivity for detection of medial and lateral wall orbit was 56% and 88%, respectively [32, 34], whereas the least specificity was 90% and 87%, respectively [31, 32]. The overall accuracy for the detection orbital wall fracture was 90–100% [9].

Fractures of the inferior orbital rim are easily detected by ultrasonography (35). The ultrasonography was performed with a 7.5 MHz curved array transducer with a sensitivity of 94% and a specificity of 92%, and a diagnostic accuracy of 92%. The positive predictive value (PPV negative predictive value (NPV) were 91% and 92%, respectively. We tested the performance of linear-probe ultrasound which is always available in ED and used for trauma patients. The diagnostic results in this study showed that a linear-probe ultrasound is not inferior to a curved array transducer; its sensitivity (92.9%), specificity (90%), PPV (86.7%), NPV (94.7%) and accuracy (79%) were significantly better than those of plain film X-ray.

This study compared the results obtained by a radiologist and senior plastic resident as examiners. Although ultrasonography is operator dependent and requires experienced personnel, there is very good interobserver reliability. Senior plastic resident may represent a non-boarded radiologist or general practitioner who practices in the ED and can use a linear probe to screen patients with clinically suspected infra orbital rim fracture during primary or secondary survey of advance trauma life support process. It has the advantage of screening in short duration of time and can performed in emergency room. The resident who participated in the study has said that they feel more confident when they have more experience while they performed using ultrasonography.

From the results, it could be concluded that ultrasonography is also helpful in screening and diagnosis of infraorbital rim fracture in some situations. It is particularly useful in a rural hospital where a confirmed diagnosis is necessary before transferring the patient to a hospital with government insurance. If the focus is only on inferior orbital rim, ultrasonography can replace plain film X-ray. Ultrasonography also can measure the gap and stepping of fracture site that can help to assess bone displacement. However, weakness of linear probe cannot detect the orbital floor fracture when compared with curvilinear array endocavity ultrasound, but it is rarely available in the emergency room. In severe orbital trauma or injuries to the skull and central nervous system, CT remains the standard option, because intra-cranial injuries and compressions of the optic nerve cannot be adequately evaluated by ultrasonography. Our study limitation is that we did not use intraoperative findings as a gold standard. In this regard, further studies may be required to provide acceptable results using US methodology.

## Conclusions

Linear probe ultrasonography has better diagnostic performance and reliability than plain film X-ray and can be used as an alternative investigation tool to CT in inferior orbital rim fracture.

## Data Availability

The datasets used and/or analysed during the current study are available from the corresponding author on reasonable request.

## Abbreviations

CT: Computerized tomography
PPV: Positive predictive value
NPV: Negative predictive value
